# The effect of phasic versus combined neuromuscular electrical stimulation using the StimaWELL 120MTRS system on multifidus muscle morphology and function in patients with chronic low back pain: a randomized controlled trial protocol

**DOI:** 10.1186/s12891-022-05578-1

**Published:** 2022-07-01

**Authors:** Maryse Fortin, Daniel Wolfe, Geoffrey Dover, Mathieu Boily

**Affiliations:** 1 Department Health Kinesiology and Applied Physiology, Concordia University, 7141 Sherbrooke Street W, SP-165.29, Montreal, QC H4B 1R6 Canada; 2 PERFORM Centre, Concordia University, Montreal, QC Canada; 3 Centre de Recherche Interdisciplinaire en Réadaptation (CRIR), Montreal, QC Canada; 4 Department of Diagnostic Radiology, McGill University Health Centre, Montreal, QC Canada

**Keywords:** Low back pain, Electrical stimulation therapy, Multifidus muscle, MRI, Ultrasound

## Abstract

**Background:**

Neuromuscular electrical stimulation (NMES) is used to improve muscle strength clinically when rehabilitating various musculoskeletal disorders. However, the effects of NMES on muscle morphology and function in individuals with non-specific chronic low back pain (CLBP) have scarcely been investigated. Although research links deficits in the paraspinal musculature with subjective reports of pain and disability, it is unknown if treatment with NMES can help reverse these deficits. Therefore, the primary aim of this study is to compare the effects of two muscle therapy protocols with a medium-frequency electrotherapy device (the StimaWELL 120MTRS system) on multifidus muscle morphology and function in CLBP patients. The secondary aims are to determine the effects of these protocols subjective reports of pain intensity, pain interference, disability, and catastrophizing.

**Methods:**

A total of 30 participants with non-specific CLBP, aged 18–60, will be recruited from local orthopedic clinics and databases. Participants will be randomized (1:1) to either the phasic or combined (phasic + tonic) muscle therapy protocols on the StimaWELL 120MTRS system. Participants will undergo 20 supervised electrotherapy treatments over a 10-week period. The primary outcomes will be multifidus morphology (e.g. cross-sectional area (CSA), fat infiltration) and function (e.g., contraction measured via %thickness change from a rested to contracted state, and stiffness at rest and during contraction). Secondary outcomes will include pain intensity, interference, disability, and catastrophizing. Both primary and secondary outcomes will be obtained at baseline and at 11-weeks; secondary outcomes measured via questionnaires will also be obtained at 6-weeks, while LBP intensity will be measured before and after each treatment. Paired t-tests will be used to assess within-group changes for all primary outcome measures. A two-way repeated-measures analysis of variance will be used to assess changes in secondary outcomes over time.

**Discussion:**

The results of this trial will help clarify the role of medium-frequency NMES on lumbar multifidus morphology and function.

**Trial Registration:**

NCT04891692, registered retrospectively on May 18, 2021.

## Background

Chronic low-back pain (CLBP) is the leading cause of years lived with disability globally[1]. It poses a significant financial burden to health-care systems: in Canada alone, healthcare costs for low back pain range from $6 to $12 billion annually[2, 3]. The vast majority of CLBP cases are of unknown etiology, meaning that a link between pain and a specific pain-generating structure cannot be established[4]. Nevertheless, research suggests that CLBP patients have deficits in the lumbar multifidus[5], a local back muscle which helps with spinal stability and load transfer[6].

There is evidence of morphological and functional changes in the multifidus in individuals with CLBP, including increased fat infiltration[7], increased stiffness at rest[8, 9], and decreased cross-sectional area (CSA)[10], which is negatively correlated with the muscle’s ability to produce force[7]. Murillo et al. found increased stiffness in the fibers of the superficial multifidus (SM), as well as decreased stiffness with contraction, in individuals with CLBP compared with healthy controls.[9] In addition, people suffering from LBP have a harder time voluntarily activating the multifidus.[11] There are theoretical rationales behind these changes. A decrease in CSA occurs with disuse and atrophy, while fatty and fibrotic infiltration reduces muscle quality, impacting the contractile output of the muscle.[12] Abnormalities in multifidus stiffness might be related to 1) elevated sympathetic nervous system activity leading to increased muscle tone in the superficial multifidus, and 2) a shift in multifidus fiber type from type II to the stiffer type I fibers[9]. Given the link between CLBP and multifidus muscle deficits, interventions that can reverse these deficits should have promising clinical outcomes.

There is mixed evidence as to whether exercise therapy, a common conservative CLBP intervention, can induce morphological and functional changes in the lumbar multifidus. Recent studies suggest that motor control exercises[13],stabilization exercises[14, 15], and high-load exercises[13](i.e., deadlift) may be effective at improving lumbar multifidus stiffness. On the other hand, a study using a machine-based, resistance exercise program saw no effects on multifidus CSA or fat infiltration[16]. Regardless, exercise therapy is not always a feasible modality. Individuals with fear-avoidance behaviors may be unwilling to engage or have reduced compliance with regards to exercise, while others with reduced mobility and function may find exercise interventions unsustainable, if not impossible. If other modalities are shown to be effective at improving lumbar multifidus morphology and function, it will alleviate the burden on exercise therapy to fulfill this role, and a wider scope of CLBP patients will benefit from conservative treatment.

Electrical stimulation therapy is a treatment modality commonly used to treat pain and muscle dysfunction. Specifically, clinicians use neuromuscular electrical stimulation (NMES) to restore strength and muscle function following atrophy or loss of neuromuscular control. NMES preferentially stimulates alpha motor neurons, causing involuntary muscular contraction. Over time, the treatment can help individuals relearn how to voluntarily contract the target muscle and improve patient outcomes[17]. NMES has been used most extensively on the quadriceps muscle, with evidence that it improves quadricep cross-sectional area following atrophy, with and without voluntary contraction[18]. However, research into its effect on lumbar multifidus morphology, as assessed with MRI or ultrasound, is very limited. Coghlan et al. (2011) reported that a 6-week NMES intervention improved resting, but not contracting, multifidus thickness in CLBP patients.[19] A recent study investigating the effect of a 4-week Russian current intervention in young women with CLBP found a non-statistically significant increase in lumbar multifidus thickness, with medium effect sizes.[20] The results from NMES interventions on CLBP patient outcomes more broadly are also mixed. Hicks et al. (2016) trial found that combined trunk muscle training with NMES was more effective than a passive control intervention (heat + ultrasound + massage) at improving performance-based and self-rated function in older adults with CLBP.[21] Another study investigated the effect of a 4-week NMES protocol on CLBP patients saw no improvement in disability compared with matched controls[22], while Alrwaily et al. (2019) reported no additional benefit of NMES on self-reported pain and disability, fear-avoidance beliefs, and paraspinal muscle strength following a stabilization exercise program and combined stabilization + NMES program in patients with CLBP patients.[23]

In all but one of the studies cited above, researchers used traditional NMES protocols, in which the current is delivered at ranges of 10–100 Hz[24].This frequency range is sometimes described as being uncomfortable for patients, because skin impedance to current is thought to be inversely proportional to stimulation frequency[17, 18]. Medium-frequency electrical stimulation therapy, delivered at ranges of 1-10 kHz, is considered by advocates to penetrate the skin more easily than low-frequency stimulation. Proponents claim this makes it both more effective at muscle stimulation, and more comfortable for patients. Tolerance to treatment is critical because patients who tolerate higher current intensities experience greater real-time multifidus thickening[25, 26], which may lead to hypertrophic and strength gains over time. To date, the effects of medium-frequency electrical stimulation therapy on multifidus muscle morphology and function in CLBP have not been investigated. Therefore, the primary aim of this study is to investigate the effects of a 10-week muscle therapy intervention using the StimaWELL 120MTRS system (a medium-frequency electrotherapy device) on multifidus muscle morphology and function. Secondary aims are to investigate its effect on pain intensity, pain interference, pain catastrophizing, and disability.

## Methods

### Study design

**Fig. 1:**
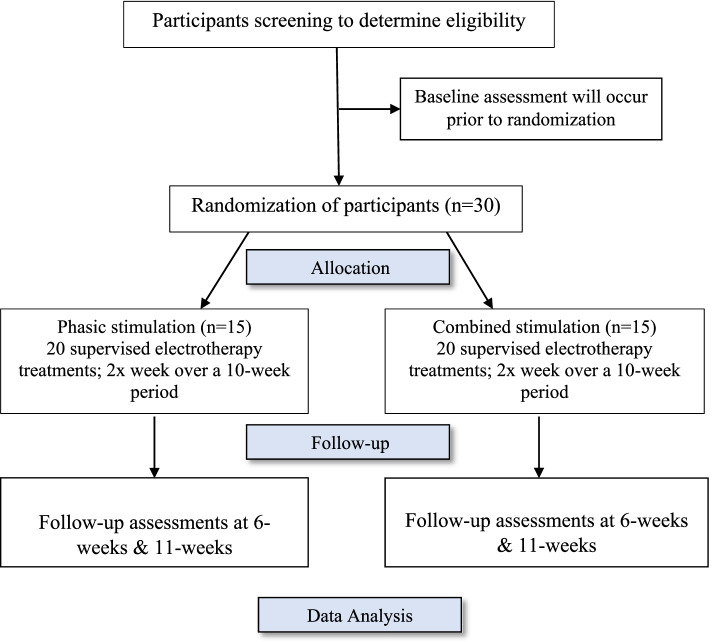
Consort Flow Diagram

The proposed study is a two-arm randomized controlled trial with test–retest design. 30 participants will be recruited and randomized into one of two muscle therapy protocols for the lumbar spine: the ‘phasic’ group (*n* = 15) or the ‘combined’ group (*n* = 15) (Fig. 1).

### Study setting

This trial will be conducted at the PERFORM Centre (Concordia University, Montreal, QC, Canada). The proposed trial was developed in accordance with the SPIRIT guidelines and approved by the Central Ethics Research Committee of the Quebec Minister of Health and Social Services (#CCER-20–21-07). All participants will be required to sign an informed consent form prior to beginning the study.

### Participant recruitment

Participants will be recruited by students and clinicians affiliated with the Quebec Low Back Pain Consortium, through the PERFORM Centre’s website and mailing list, over social media (Facebook, Instagram), and through word-of-mouth. Individuals affiliated with the Quebec Low Back Pain Consortium who agree to be contacted for studies will receive either a telephone call or email explaining the study aims and procedure. All prospective participants will undergo a preliminary phone screening to verify eligibility. Those who pass the phone screen will be invited to the PERFORM Centre for a neurological screen, and a trial visit with the StimaWELL 120MTRS system.

### Participants

#### Inclusion Criteria

Participants must meet all the following criteria for inclusion:

- Chronic non-specific LBP (> 3 months), defined as pain in the region between the lower ribs and gluteal folds, with or without leg pain.
- Aged between 18 to 60 years old.
- English or French speakers
- Have at least score of ‘moderate’ on the Modified Oswestry Disability Index (ODI) (to ensure a minimal important change [MIC] is detectable)[27].
- Able to undergo MRI exam

#### Exclusion Criteria

Participants will be excluded if they meet any of the following criteria:

- Currently undergoing or having received physical therapy treatment in the previous month
- Consistent motor control training for the low back and / or consistent weightlifting, powerlifting, bodybuilding, or strongman training in the previous 6 weeks (resistance training is a potential confounding variable; however, muscle strength and size decreases 2–6 weeks following the cessation of resistance training in both trained and un-trained individuals[28, 29].)
- History of lumbar surgery
- Presence of positive lumbosacral dermatomes or myotomes
- Presence of disease which could affect the stiffness of muscle tissue (collagen tissue disease, hemiplegia, multiple sclerosis, blood clots)
- Presence of systemic disease (cancer, metabolic syndrome)
- Presence of spinal abnormality (spinal stenosis, fracture, infection, tumor**,** or lumbar scoliosis greater than 10 degrees)
- BMI > 30 (subcutaneous fat attenuates ultrasound signal and can invalidate measurements in obese individuals)[30].
- Presence of cardiac arrhythmia
- Pregnant and breastfeeding women
- Individuals with epilepsy
- Individuals at risk for serious bleeding
- Individuals with pacemakers or metal implants
- Individuals with aneurysms or heart valve clips
- Individuals who have taken prescribed muscle relaxants more than once a week in the previous month

### Randomization

Participants will be randomized to treatment groups (1:1) using consecutively numbered sealed opaque envelopes (e.g., computer-generated randomization sequences) created by an individual not involved in the study.

### Personnel

A PhD student (who is a certified athletic therapist), not blinded to group allocation, will conduct most in-person activities, including neurological testing, ultrasound evaluations, and intervention administration. In Canada, certified athletic therapists are health-care practitioners who specialize in the prevention, assessment, and treatment of musculoskeletal injuries. A PERFORM Centre technician will administer the MRI exam. Participant recruitment and preliminary screening will be conducted jointly between the PhD student and a research assistant working for this study’s primary investigator.

### Intervention

All research activities will take place at the PERFORM Centre, Concordia University. This center houses 8000m^2^ of laboratories, assessment suites, and lifestyle intervention spaces. It is equipped with the instruments needed to assess this study’s primary outcomes (MRI, Ultrasound), as well as space for conducting the intervention.

**Fig. 2:**
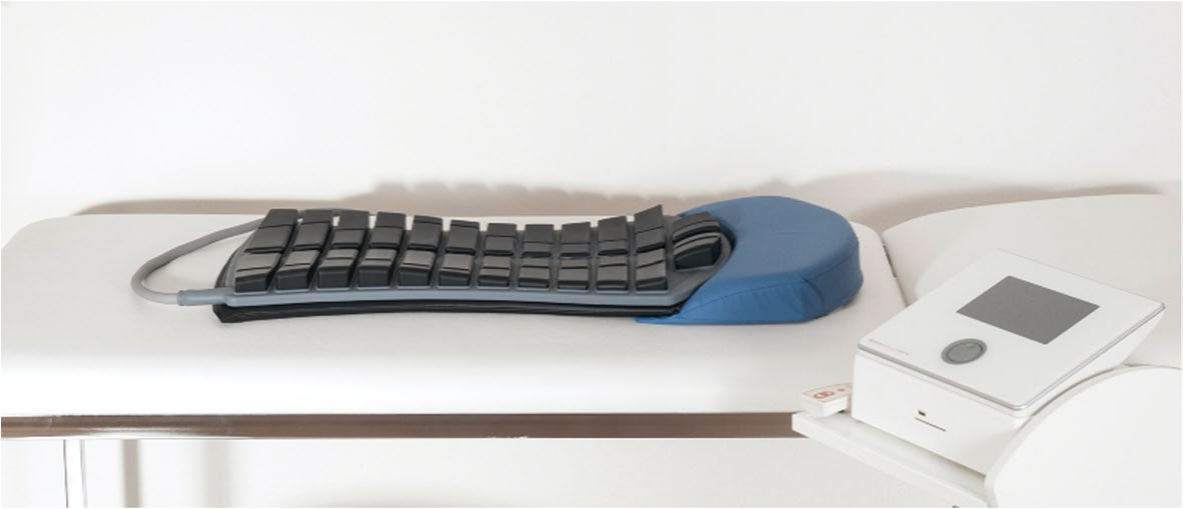
StimaWELL 120MTRS system

All participants will receive treatment with the StimaWELL 120MTRS system (schwa-medico, Germany) (Fig. 2). The StimaWELL 120MTRS system is a pre-modulated IFC (interferential current) electrotherapy device. It delivers current across up to 12 channels and offers preset pain and muscle therapy programs. The device also heats up to 40 °C.

#### Phasic intervention group

Participants in this group will receive therapy at the StimaWELL 120MTRS system’s setting for phasic muscle stimulation of the lumbar spine (3 kHz, modulation 50 Hz). Tonic muscles are postural muscles composed of a majority of type I fibers, while phasic muscle are prime movers composed of a majority of type II fibers. We are choosing the phasic setting because some research has shown an increase in stiffness in the superficial multifidus (SM) in CLBP patients9, which contain a greater proportion of type II fibers in healthy individuals. The increase in stiffness could reflect a shift towards type I fiber composition in the SM in CLBP patients. Therefore, parameters which selectively target type II fibers might help reverse this process. During the initial calibration and throughout the treatments, the current intensity will be increased so that participants feel a strong but comfortable contraction. This standard of current intensity will always be maintained, although the actual output may vary over the course of treatment.

#### Combined (tonic and phasic) group

Participants in this group will receive therapy at the StimaWELL 120MTRS system’s setting for combined stimulation (3 kHz, modulation 4 Hz and 50 Hz). We are investigating the efficacy of the combined setting since 23–46% of multifidus fibers are type II fibers[31]. A treatment that targets these fibers should also leads to improvements in multifidus physiology. During the initial calibration and throughout the treatments, the current intensity will be increased until participants feel a strong but comfortable contraction. This standard of current intensity will always be maintained, although the actual output may vary over the course of treatment.

#### Timeline

The intervention period will last 10 weeks, with treatments occurring twice a week for both groups. There is evidence that a minimum of five weeks of training are needed to induce muscular hypertrophy[32]. The treatment will last 20 min for the first 3 weeks, 25 min for the second 3 weeks, and 30 min for the last 4 weeks; these times are in line with norms for NMES interventions[25]. Additionally, participants will come to the PERFORM Centre for two pre-intervention visits (trial visit & questionnaire completion, MRI and ultrasound evaluation), and one post-intervention visit (questionnaire completion, MRI and ultrasound evaluation) for a total of 23 visits (Table 1).

**Table 1:** Schedule of enrolment, interventions, and assessments

	Enrolment	Baseline	Intervention			Post-Intervention
TIMEPOINT	*-t*_*1*_	0	*t*_*1 – 10*_	*t*_*11*_	*t*_*12-20*_	*t*_*21*_
ENROLMENT:						
Eligibility screen	X					
Informed consent		X				
Neurological screen		X				
Trial of wave-mat		X				
Allocation		X				
INTERVENTIONS:						
Phasic Group			X	X	X	
Combined Group			X	X	X	
ASSESSMENTS:						
NPRS Full		X		X		X
NPRS Pre-Post			X		X	
ODI	X	X		X		X
BPI		X		X		X
PCS		X		X		X
MRI		X				X
Ultrasound		X				X

-**t**_1_ = pre-intervention; 0 = baseline visit; ***t***_***1 – 10***_ = treatments 1–10; ***t***_***11***_ = treatment 11; ***t***_***12-20***_ = treatments 11–20; ***t***_***21***_ = post-intervention visit

### Primary outcome measures

#### MRI assessment of multifidus morphology

All participants will undergo a lumbosacral MRI evaluation using the PERFORM Centre’s 3-T GE machine to assess multifidus muscle CSA and fat infiltration. MR imaging will be collected using a standard phased-array body coil with 4-mm slice thickness, 180-mm2 field of view and 512 × 512 matrix. Quantitative multifidus muscle measurements will be obtained from axial T2-weighted images, bilaterally at the L4-L5 and L5-S1 spinal levels, which are the most relevant levels for spinal pathologies. The multifidus muscle CSA will be measured manually at both levels on multiple slices to calculate the summative 3D volume; cross-sectional area measurements have been widely used to assess muscle size and this technique is very reliable (ICCs:0.97–0.99)[33]. Multifidus muscle composition (e.g., fatty infiltration) will be assessed using IDEAL (Lava-flex, 2 echo sequence) fat and water images by calculating percent-fat signal fraction at each spinal level according to the following equation: %FSF = (Signal_fat_/[Signal_water_ + Signal_Fat_] × 100)[34].

#### Ultrasound assessment of multifidus muscle function

The PERFORM Centre’s Aixplorer ultrasound unit (Supersonic Imagine, Aix-en-Provence, France) will be used to assess multifidus muscle contraction and stiffness. First, participants will be placed in a prone position, on a therapy table, with a pillow placed under their abdomen to minimize lumbar lordosis (e.g., maximum of 10° measured with an inclinometer) and instructed to relax the paraspinal musculature. The spinous process of L5 will be palpated and marked on the skin with a pen prior to imaging. Acoustic coupling gel will be applied to the skin and the ultrasound transducer placed longitudinally along the midline of the lumbar spine to confirm the location of the L5 level. The multifidus muscle will be imaged bilaterally, in the parasagittal section, allowing for the visualization of the L5/S1 zygapophyseal joints. Multifidus muscle contraction, expressed as the % thickness change from a rested to contracted position, will be assessed via contralateral arm lifts while holding a small handheld weight (e.g., 1.5 to 3 pounds) based on the participant’s body weight. Participants will be instructed to raise the loaded arm 5 cm off the examination table with the shoulder in 120° of abduction and elbow 90° of flexion, following a deep inhalation and exhalation. Images will be taken after the loaded arm has been held for 5 s. The handed weight is designed to load the multifidus muscle to approximately 30% of maximal voluntary isometric contraction. Measurements will be obtained at L4-L5 and L5-S1 and repeated 3 times, both at rest and during contraction on each side. The average of 3 thickness ratios ([thickness contracted – thickness rest / thickness rest] × 100) will be calculated in used in the analysis. This technique is valid and reliable[35, 36].

The same position and procedure will be used to assess multifidus muscle stiffness with shear-wave elastography. This technique is based on a compressive wave that propagates within the tissue, allowing for the calculation of tissue shear wave modulus while rendering a quantitative color-coded map of tissue elasticity. Participants will be lying prone on the therapy table for 5 min before the lumbar multifidus is imaged at rest and during sub-maximal contractions while performing the same task as described above. Three repetitions will be performed on each side (both at L4-L5 and L5-S1) and the average shear wave modulus will be used for analysis. Finally, multifidus muscle stiffness will also be examined in a standing position when the muscle is naturally contracting in a stabilizing role. Participants will be asked to stand barefoot on the floor with their arms relaxed on each side. To achieve a habitual standing posture, they will be instructed to march on a spot for a few seconds and remain on the position where their feet landed. Resting shear wave modulus measurements will be acquired as described above and obtained in the standing fundamental position (e.g., arms resting naturally on each side of the body). Again, three measurements will be obtained on each side (both at L4-L5 and L5-S1) and the average shear wave modulus will be used in the analysis. This technique is valid and reliable[37–39].

### Secondary outcome measures

#### Pain intensity

Pain intensity will be measured with the Numerical Pain Rating Scale (NPRS). The NPRS measures pain intensity on a scale of 0—10, with 0 indicated no pain, and 10 indicating the worst pain imaginable. Changes of 2 or more points are clinically significant[40]. At baseline, midpoint, and endpoint, participants will be asked to rate the following using the NPRS: current low back pain, current leg pain, best and worst pain low back pain over the previous week, pain sitting and with movement over the past 24 h. Additionally, participants will be asked to rate their current low back pain prior to, and at the end of each treatment.

#### Pain interference

Pain interference will be measured using the Brief Pain Inventory, interference subsection (BPI). The BPI-I is a 7-item questionnaire that measures how pain interferes with activities of daily living. Each item is rated from 0–10. Higher scores indicate greater interference.

#### Disability

Disability will be assessed using the Oswestry Disability Index (ODI, is used to measure disability in relation to LBP. It is a 10-item scale in which each item is rated from 0–5. Higher scores indicate greater disability, and changes of > 10% are clinically significant[27].

All three questionnaires are valid and reliable measures of LBP and function[27, 40, 41].

### Possible effect modifiers

#### Catastrophizing

Pain catastrophizing will be assess using the Pain Catastrophizing Scale (PCS). The PCS is a 13-item questionnaire that assesses the participant's level of catastrophizing. Each item is rated from 0–4 for a possible total of 52. Higher scores indicate greater catastrophizing, with scores above 30 being clinically significant[42].

Participants will complete the BPI, ODI, and PCS at baseline, midpoint (6-weeks), and post-intervention (11-weeks).

### Procedure

#### Trial visit

Participants who pass the phone screening will be invited for a trial visit. Upon arrival, the PhD student will ask them to sign a consent form. Second, the PhD student will assess lumbosacral dermatomes and myotomes to ensure the absence of nerve root compression. Third, participants will fill out a sociodemographic questionnaire, as well as questionnaires regarding secondary outcomes (see above). Fourth, participants will receive a 10-min trial treatment with the StimaWELL 120MTRS system at its setting for ‘combined’ lumbar muscle therapy.

#### Wave-mat calibration

The StimaWELL 120MTRS system delivers current at a given intensity across up to 12 channels. However, in the presence of pain or injury the current may not be felt equally across channels. Therefore, prior to the trial, the wave-mat will be calibrated to ensure that the current is equally felt across all channels. A paper towel will be sprayed with warm water and laid over top of the wave-mat. Participants will be asked to remove their top (and unbuckle their bra, if applicable), lie supine on the towel with knees bent, and lower the top of their underwear so that their coccyx is touching the lowest channel. A blanket will be provided for privacy. During the initial phase of calibration, the current travels the length of the wave-mat, from bottom to top, in repetitive fashion. The current will be increased to tolerance (e.g., until participants feel a strong, but non-painful, sensation). Then, the current will be adjusted, channel by channel (from bottom to top), to ensure that each channel is set to the appropriate intensity. Once this is complete, the current will be adjusted from side-to-side, in groups of two channels (from bottom to top), to ensure the intensity is the same from one side to another at a given level. This completes the calibration. This process will be repeated prior to the start of participants’ 5^th^, 9^th^, 13^th^, and 17^th^ treatments, to account for unilateral and segmental adaptations, and to ensure the multifidus is appropriately stimulated.

### Data monitoring

#### Adverse events

The occurrence of adverse events in response to the treatment (ex: muscle soreness, temporary increase in pain or stiffness) will be monitored by the PhD student during the intervention using open-ended questions.

#### Co-interventions

Participants will be asked about co-interventions (physiotherapy visits, medication, exercise, injection) during each visit. Any co-interventions will be recorded.

Treatment modification3.

During all sessions, participants will be provided a remote to increase or decrease the intensity of the current to tolerance, as needed.

### Sample size calculation

While the effect of NMES on multifidus muscle morphology and function has not been thoroughly investigated in subjects with CLBP, previous reports showed significant improvements in multifidus thickness at rest and during contraction with medium to large effect sizes following a 6-week[19] and single NMES session[43], respectively. Based on these studies, we used a mean effect size of 0.9 to calculate our sample size at a level of confidence of 0.05 and 80% power. Accordingly, a sample size of 12 participants in each group was needed. We increased the sample size to 15 participants in each group to account for potential drop-out.

### Statistical analysis

We will perform an exploratory data analysis on participants’ sociodemographic characteristics and to verify normality assumptions. Paired t-tests or Wilcoxon sign-rank tests will be used assess within-group changes, pre-to-post intervention, for all primary outcome measures. Independent t-tests or Mann Whitney-U tests will be used to assess between-group changes, pre-to-post intervention, for all primary outcome measures. We will use either a two-way repeated measures ANOVA or Kruskall-Wallis tests to determine changes over time for secondary outcome measures. For all tests, statistical significance will be set at *p* < 0.05.

## Discussion

CLBP is a major health concern in Canada. It imposes a significant cost on our healthcare system and is detrimental to our population’s quality of life. Additionally, CLBP is linked to morphological and functional deficits in the multifidus muscle. To date, very little research has been done investigating the utility of electrotherapy in improving multifidus muscle thickness and CSA in CLBP patients. To the best of our knowledge, no research has been done into its effects on multifidus stiffness and fat infiltration in this population. This clinical trial constitutes novel research in two ways. First, it will help fill a gap in orthopedic NMES research, the majority of which has been focused on lower extremity use. Second, it will add to the body of research investigating the role of paraspinal morphology and function in CLBP. Moreover, if this treatment protocol is effective at improving multifidus morphology and function, our findings may lead to an improvement in the overall efficiency of CLBP treatments.

This study has several limitations. Firstly, our treatments are limited to twice per week. Many NMES protocols recommend treatments up to 6–7 times per week with portable units; unfortunately, this is not possible with the StimaWELL 120MTRS system. We have chosen to treat twice a week to maximize patient compliance, and an intervention period of 10 weeks was chosen to ensure an adequate number of exposures. Second, this study excludes individuals with a BMI > 30. We are taking this step because the presence of excessive adipose tissue and intramuscular fat can affect the validity of the shear-wave measurements, as fat attenuates the signal propagating from the ultrasound soundhead. We are also excluding individuals over 60 years old because intramuscular fat naturally increases with age, and this may confound our results. Therefore, the results of this trial may not be generalizable to adults over 60 years old, and those with a BMI > 30.

## Data Availability

Not applicable.

## Abbreviations

CLBP: Chronic low back pain
NMES: Neuromuscular electrical stimulation
CSA: Cross-sectional area
BMI: Body mass index
SM: Superficial multifidus
